# Pandemic Risk Assessment for Swine Influenza A Virus in Comparative In Vitro and In Vivo Models

**DOI:** 10.3390/v16040548

**Published:** 2024-03-31

**Authors:** Ian Padykula, Lambodhar Damodaran, Kelsey T. Young, Madelyn Krunkosky, Emily F. Griffin, James F. North, Peter J. Neasham, Vasilis C. Pliasas, Chris L. Siepker, James B. Stanton, Elizabeth W. Howerth, Justin Bahl, Constantinos S. Kyriakis, Stephen Mark Tompkins

**Affiliations:** 1 Center for Vaccines and Immunology, University of Georgia, Athens, GA 30602, USA; 2 Department of Infectious Diseases, University of Georgia, Athens, GA 30602, USA; 3 Emory-UGA Centers of Excellence for Influenza Research and Surveillance (CEIRS), Athens, GA 30602, USA; 4 Department of Pathology, University of Georgia, Athens, GA 30602, USA; 5 Department of Pathobiology, Auburn University, Auburn, AL 36849, USA

**Keywords:** humans, swine, mice, ferrets, pandemics, risk assessment, influenza A virus, epithelial cells, whole genome sequencing, models, animal

## Abstract

Swine influenza A viruses pose a public health concern as novel and circulating strains occasionally spill over into human hosts, with the potential to cause disease. Crucial to preempting these events is the use of a threat assessment framework for human populations. However, established guidelines do not specify which animal models or in vitro substrates should be used. We completed an assessment of a contemporary swine influenza isolate, A/swine/GA/A27480/2019 (H1N2), using animal models and human cell substrates. Infection studies in vivo revealed high replicative ability and a pathogenic phenotype in the swine host, with replication corresponding to a complementary study performed in swine primary respiratory epithelial cells. However, replication was limited in human primary cell substrates. This contrasted with our findings in the Calu-3 cell line, which demonstrated a replication profile on par with the 2009 pandemic H1N1 virus. These data suggest that the selection of models is important for meaningful risk assessment.

## 1. Introduction

The ability of influenza A viruses of various genetic backgrounds to infect swine is of significant concern to public health. Swine have been proposed as key intermediate hosts in the adaptation of avian influenza viruses to mammalian species, but they are also susceptible to infection with human influenza isolates. As multiple influenza viruses infect a single individual, they have the potential for reassortment of gene segments that can result in a novel virus to which the host has little or no immunity. This was exemplified in the 2009 H1N1 pandemic, in which gene segments from human, avian, and swine origins reassorted to create a novel, antigenically shifted virus that rapidly spread across the globe [1,2].

Currently endemic in swine populations are influenza strains belong to the H1N1, H1N2, and H3N2 subtypes [3]. In the U.S., human seasonal influenza spilled into commercial swine populations in the early 2000s, reassorting with circulating swine influenza A viruses to create a lineage of H1N2 that possesses external proteins of human seasonal influenza origin with a swine internal gene cassette [4,5]. This lineage, designated 1B.2.2 or δ-2, has continued to diversify within swine populations since this point of introduction. This has resulted in an increasingly dominant endemic virus within commercial herds as well as several human cases of H1N2 variant (H1N2v) viruses [6]. The ability of these swine viruses to spill back into human populations suggests there is limited immunity from cross-protective antibodies elicited by human seasonal influenza [7]. As such, increased surveillance efforts have been focused on these viruses.

In order to properly assess the risk potential of emerging influenza strains, a framework has been established by the Center for Disease Control and Prevention, the Influenza Risk Assessment Tool (IRAT) [8]. The tool allows for a systematic evaluation of emerging zoonotic influenza strains and a baseline comparison for prioritization and allocation of funding. By analyzing a given virus according to three main criteria: viral properties, host properties, and epidemiological factors, the tool seeks to answer two key questions. The first is that of emergence, that is, what is the risk of a novel influenza strain being capable of sustained human-to-human transmission. The second question is that of the impact of viral infection on public health if sustained transmission is possible. The three main criteria of the IRAT are broken down into a total of 10 elements, several relating to a virus’s ability to transmit and cause disease within animal species as well as humans. The usage of animal models to assess risk to human populations is quite varied and involves additional cost and risk to investigators. Several in vitro model systems have also been used to determine the susceptibility of human respiratory tissues, with increasingly complex culture systems allowing for growth and differentiation of primary epithelial cell cultures from host animals that approximate the in vivo environment [9,10,11,12,13].

We isolated an influenza A virus from a lethal case of swine influenza, A/swine/Georgia/A19-27480/2019 (H1N2; GA/19), that we then assessed in multiple models of infection to determine the extent of pathogenesis in its host species as well as the transmission potential to humans. Phylogenetic analysis determined the isolate as belonging to the 1B.2.2 lineage, with genetic relatedness to Midwest swine influenza viruses but also H1N2v viruses. Infections in mice, swine, and ferrets showed the ability of the virus to infect multiple animal species and transmit to naïve contact animals. We further investigated risk to humans through infections in tissue culture systems, including Calu-3 cells and primary respiratory epithelial cells from human donors. Despite the human origins of both these substrates, results presented differing susceptibility of human tissues to the swine isolate that was dependent only on the model used.

## 2. Materials and Methods

### 2.1. Origin of the Virus and Its Isolation

A 6-month-old Hampshire cross market gilt was submitted for necropsy to the Athens Veterinary Diagnostic Laboratory, College of Veterinary Medicine, UGA, after a sudden and severe illness of unknown etiology [14,15]. The animal was a 4-H show pig with a recent history of travel to an event. Upon necropsy, there was severe cranioventral consolidation with necrotizing bronchointerstitial pneumonia and ulcerative tracheitis. Confirmatory fluorescent antibody testing and immunohistochemistry (IHC) on lung tissue diagnosed influenza A bronchitis. Bronchiolar sections of lung tissue weighing approximately 100 g were homogenized, and the resulting homogenate was passed through a 0.45 µm nylon mesh filter. The homogenate was then used to inoculate flasks of MDCK cells. The virus was passaged twice blindly and then plaque purified. The plaque-purified virus was used in further animal and in vitro infection studies.

### 2.2. Phylogenetic Analysis

Viral sequence data were generated using a MinION platform, as previously described [15]. MinION reads were assembled using IRMA v 0.6.7 [16] and validated by Illumina sequencing, as previously described [17]. Sequences are publicly available in GenBank, under BioProject PRJNA600894, NCBI accession numbers PP554726, PP554727, PP554728, PP554729, PP554730, PP554731, PP554732, and PP558395. To investigate the potential for the isolates’ involvement in human–swine transmission and the virus’s evolutionary history, the consensus sequences for the HA protein (H1) and NA protein (N2) were used in phylogenetic analyses. Nucleotide sequence data for viruses isolated in North America were collected from the NCBI Influenza Virus Resource (date range: 6/6/2014 through 7/2/2019) and grouped into separate datasets for human H1 (n = 5761), swine H1 (n = 4868), human N2 (n = 10,199), swine N2 (n = 3958), human M (n = 5863), and swine M (n = 3341) [18].

To determine potential human–swine transmission associated with the swine isolate A/swine/Georgia/A19-27480/2019 (H1N2), maximum likelihood trees were created by performing 100 bootstrap replicates of RAxML v 8.2.4 for the coding regions of each segment dataset [19]. The trees were created using a generalized time reversible nucleotide substitution model with gamma distributed rate variation among sites (GTR+ Γ4). A root-to-tip regression of the estimated trees was performed to determine the molecular clock signal of the data, and temporal outliers were identified and removed using TempEst v 1.5.3 [20]. The swine isolate showed proximity to human variant isolates, which were retained for subsequent Bayesian phylogenetic reconstructions.

Bayesian phylogenetic tree estimation was performed for a subsampled dataset subset of n = 450 taxa for swine HA and NA segment datasets using BEAST v1.10.4 [21]. The Phylogenetic Diversity Analyzer v1.0.3 was used to subsample taxa from the constructed maximum likelihood phylogeny of all available isolates by randomly subsampling each clade based on specified subsample size [22]. Six independent Markov Chain Monte Carlo (MCMC) runs for both proteins were preformed using a GTR+ Γ4 substitution model, lognormal uncorrelated relaxed clock model, and a Gaussian Markov Random Field Skyride coalescent [23,24]. Each MCMC run had a chain length of 100 million states, sampling every 10 thousand states. After removing appropriate burn-in from the beginning of the run (10%), a maximum clade credibility phylogenetic tree was generated for each segment from a posterior sampling of 9000 trees.

### 2.3. Animal Infection and Transmission Experiments

Five- to eight-week-old female DBA/2 and BALB/c mice (Jackson Labs and Envigo, respectively; n = 25 per strain) were divided into groups: infected (n = 20) and mock (n = 5). Mice were infected under isoflurane anesthesia via intranasal inoculation, with 1 × 10^5^ pfu of GA/19 virus in a 30 µL volume. Mock-infected mice were administered 30 µL PBS intranasally. Animals were observed for clinical signs twice daily and weighed daily. At 2- and 4-days post-infection (dpi), a subset of five mice from each infected group was euthanized, and their lungs were collected for virus titration. At 5 dpi, three mice from each infected group were euthanized, and their lungs were collected and perfused with neutral-buffered 10% formalin before submission for histopathological examination. The remaining mice were weighed daily until 13 dpi. All mice were euthanized at 25 dpi.

Six 6-week-old influenza virus-naïve and porcine reproductive and respiratory syndrome virus (PRRSV)-naive conventional cross-bred Yorkshire/Hampshire male and female pigs were obtained from Auburn University’s Swine Research Center (an influenza virus and PRRSV-seronegative herd) for the swine study. One week prior to infection, the study animals were treated with ceftiofur crystalline free acid (Zoetis). One day before infection, the animals were sedated with an intramuscular injection of ketamine (0.5 mg/kg), xylazine (0.5 mg/kg), and tiletamine–zolazepam (1 mg/kg), and baseline bronchoalveolar lavage (BAL) samples and blood samples were taken. The animals were then separated into two groups: infected (n = 3) and contact (n = 3). Infected animals were inoculated intranasally, with 1 mL of 1 × 10^6^ pfu/mL GA/19 virus in each nostril. Temperatures and nasal swabs were taken daily after infection. At 2, 4, and 6 dpi, infected animals were sedated, and BAL and blood samples were collected. At 3 dpi, contact animals were co-housed with the infected animals. Nasal swabs were performed daily on infected animals and on contact animals after introduction. At 13 dpi, blood samples were collected from all animals.

Ten 12-week-old ferrets were divided into two groups: infected (n = 6) and contact animals (n = 4), with equal gender distribution between the two groups. Three days prior to infection, all animals were anesthetized under isoflurane, and 3 mL venous blood draws were performed, as well as the placement of subdermal temperature transponders (BMDS) for animal identification and temperature monitoring. On day 0, six animals were anesthetized and inoculated intranasally with 1 × 10^6^ pfu of GA/19 virus in a 1 mL volume distributed equally between each naris. Nasal washes and weights were taken on infected animals one day post-infection and every other day subsequently. At 2 dpi, naïve contact animals were co-housed with infected animals in a 1:1 ratio, and nasal washes and weighing were performed, as on infected animals. At 4 dpi, two infected animals were euthanized, and tissue samples were collected from the respiratory tract. At 7 and 14 dpi, venous blood draws were performed, with animals euthanized at 14 dpi.

All animal studies were reviewed and approved by the Institutional Animal Care and Use Committee (IACUC) prior to initiation.

### 2.4. Cell Culture and Infection

Calu-3 (ATCC), normal human bronchial epithelial (Lonza, Cambridge, MA, USA), and porcine primary nasal epithelial [25] cells were cultured and differentiated at an air–liquid interface (ALI) in 12-well plates, as previously described [9,10]. In brief, cells were seeded onto collagen-coated transwells of a 12-well plate (Corning, Corning, NY, USA). Media were changed one day after seeding and every other day afterward until cells had reached confluency. Apical media were then removed from the transwell, and basolateral media were replaced with ALI media (DMEM/F-12 supplemented with 1% penicillin/streptomycin, 2% NuSerum, and 50 nM retinoic acid). On day 0, apical surfaces were washed and inoculated with GA/19 virus at an MOI of 0.01 in a 200 µL volume. All infections were run in triplicate wells. Cultures were then incubated in a humidified 5% CO_2_ incubator at 37 °C for 2 h before the inoculum was removed. At 12, 24, 48, 72, and 96 h post-inoculation, 1 mL of sterile PBS was used to wash the apical cell surface, and then, the wash was assayed for viral titers.

### 2.5. Sample Processing

Mouse lungs were individually placed in 1 mL PBS after collection and kept on ice until homogenization. Lungs were homogenized, as previously described [26], clarified by centrifugation, aliquoted, and then stored at −80 °C. Bronchioalveolar lavage (BAL) fluid was placed on ice immediately after collection and then passed through a 40-micron filter before aliquoting and freezing at −80 °C. Nasal swabs were collected using polyester swabs, placed in tubes containing 2.0 mL of phosphate-buffered saline (PBS) supplemented with 1% antibiotic-antimycotic, and placed on ice immediately after collection. Samples were then sonicated for 10 min before aliquoting and storing at −80 °C [27]. Nasal washes were performed with sterile PBS, and then, samples were placed on ice after collection, clarified, aliquoted, and then stored at −80 °C.

### 2.6. Virus Titration

For plaque assays, tenfold serial dilutions were prepared from thawed samples in serum-free MEM. An inoculum of 250 µL was placed into each well of a 12-well plate of confluent Madin–Darby canine kidney (MDCK) cells (MDCK-ATL, FR-926, International Reagent Resource, Manassas, VA, USA) and incubated in a humidified chamber at 37 °C for 1 h before a 1.2% Avicel overlay supplemented with 1× TPCK trypsin was placed on the culture. Plates were then incubated for 72 h in a 5% CO_2_ humidified chamber at 37 °C before the overlay was removed, and plates were fixed with an 80% methanol 20% acetone solution. Fixed wells were then stained with crystal violet to visualize plaques that were counted, and then, infectious virus titers were calculated.

For virus quantitation by PCR, viral RNA was extracted from nasal swab samples and tested for the matrix gene of IAV. Viral RNA was extracted using RNAzol RT. RT-PCR was performed using Taqman^®^ Fast Virus 1-Step Master Mix. A 25 µL PCR mixture containing 6.25 µL 4X Fast Virus Master Mix, 14.75 µL DNase/RNase-free distilled water, 0.5 µL of each primer (forward: AGATGAGTC TTCTAACCGAGGTCG, reverse: TGCAAAAACATCTTCAAGTCTCTG), 1 µL of probe (FAM-TCAGGCCCCCTC AAAGCCGA-BHQ), and 2 µL of the sample RNA template was prepared. Reactions were run at 50 °C for 30 min, followed by 95 °C for 15 min, followed by 40 cycles at 95 °C for 10 s, then 60 °C for 20 s. Data were acquired on the BioRad C1000 Touch Thermal Cycler, and data analysis was performed with BioRad CFX Manager (v3.1). Viral titers were calculated based upon the titration of a stock of known concentration, presented as relative expression units (REUs) compared to a negative control sample.

### 2.7. Statistical Analysis

Viral titers were analyzed using analysis of variance (ANOVA) using GraphPad Prism version 8.0.0 for Windows (GraphPad Software, San Diego, CA, USA, www.graphpad.com). A *p*-value ≤ 0.05 was considered significant.

## 3. Results

### 3.1. Phylogenetic Assessment

**Figure 1:**
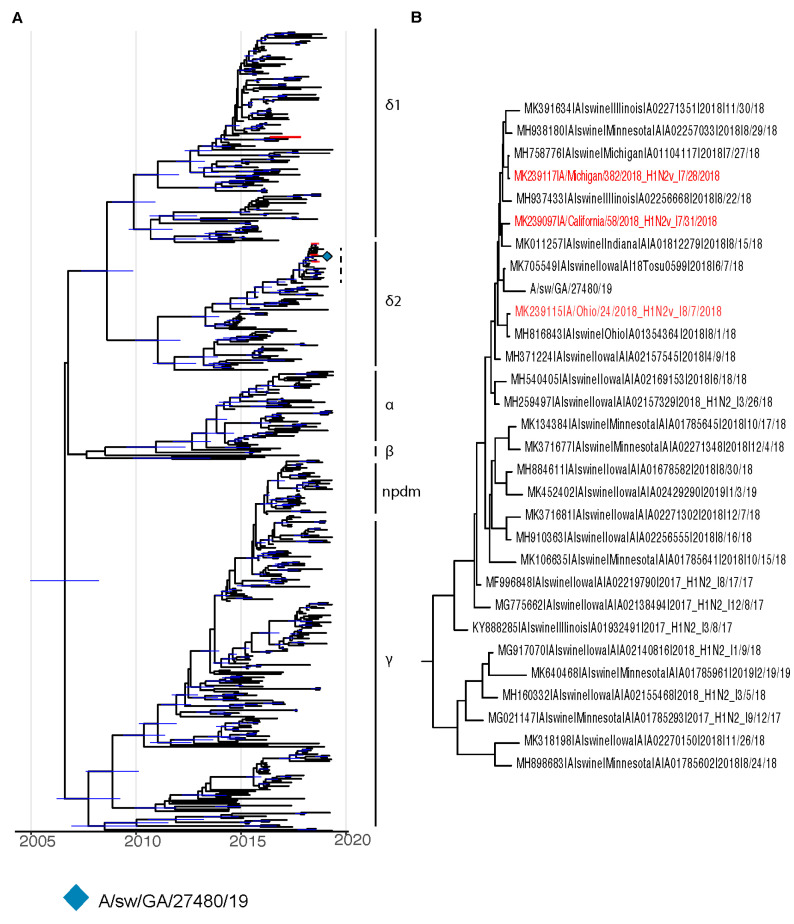
BEAST phylogenies for swine influenza H1Nx isolates collected from 2014 to 2019. (**A**,**B**) Phylogenetic reconstruction for hemagglutinin (HA) segment of swine H1Nx isolates. Nodes with a posterior support of greater than 95% are annotated with a 95% Bayesian Credible Interval in blue. Taxa for H1N2 variant isolates are colored in red.

**Figure 2:**
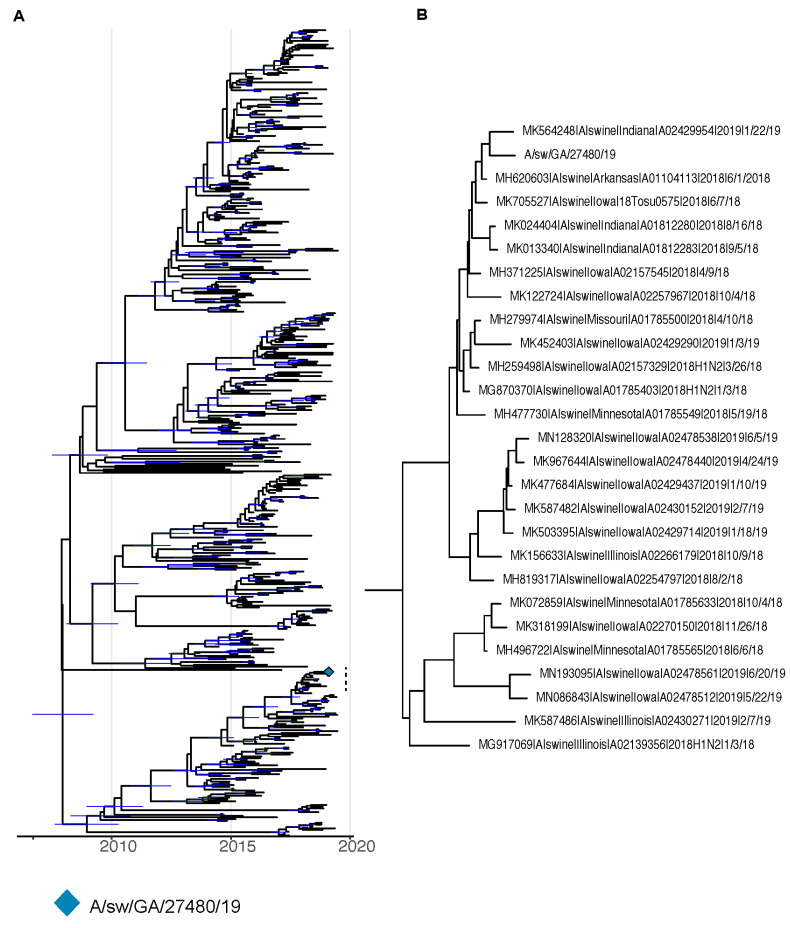
BEAST phylogenies for swine influenza A HxN2 isolates collected from 2014 to 2019. (**A**,**B**) Phylogenetic reconstruction for neuraminidase (NA) segment of swine HxN2 isolates. Nodes with a posterior support of greater than 95% are annotated with a 95% Bayesian Credible Interval in blue.

A purified viral stock was obtained from lung tissue of a diseased pig and then plaque purified before sequencing using a MinION platform. Obtained consensus sequence data identified the virus as belonging to the H1N2 subtype (1B.2.1) [15] and was confirmed by subsequent Illumina deep sequencing. We performed phylogenetic analysis of the isolate in the context of human and swine H1N2 viruses to determine the genetic distance from recent human influenza viruses of similar genetic backgrounds. Maximum-likelihood trees assembled from sequences extending five years prior to the date of sample collection revealed a significant distance between the isolate and the closest human isolate (Figure S1). However, the isolate did demonstrate a close relationship to H1N2 variant (H1N2v) cases, in which swine influenza virus resulted in limited human infections (Figure 1 and Figure 2) [28,29].

Bayesian phylogenetic trees were created for HA (Figure 1A,B) and NA (Figure 2A,B) genetic segments. The resulting trees illustrate a close genetic distance between the GA/19 isolate and contemporary swine influenza viruses circulating in the Midwest United States.

### 3.2. Viral Replication in the Murine Model

**Figure 3:**
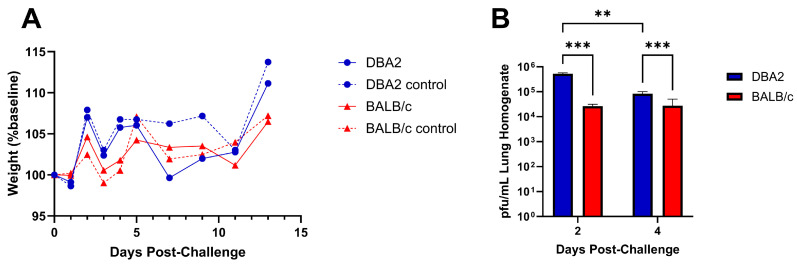
Weight change and lung virus replication following infection of DBA/2 or BALB/c mice with A/sw/GA/27480/19 (H1N2). Five- to eight-week-old BALB/c and DBA/2 mice (n = 25 mice/group) were inoculated intranasally with either 1 × 10^5^ pfu of virus in a 30 µL volume (n = 20) or with PBS (n = 5). Weight loss was tracked for 13 days post-infection (dpi) (**A**); dashed lines represent mock-infected control groups. At 2 and 4 dpi, a subset of five mice from each infected group were euthanized, and their lungs were collected. Viral titers, described as pfu/mL of lung homogenate, were determined by plaque assay (**B**). Significance values of ≤0.005 and ≤0.0005 are denoted by ** and ***, respectively. Error bars indicate mean ± SD.

Although mice do not shed influenza virus or exhibit symptomology correlating to swine or human disease, the virulence of influenza infection in mice has been shown to correlate with the severity of disease in the case of several human and swine influenza strains [15,26,28]. We assessed virus replication, pathogenesis, and clinical disease in BALB/c and DBA/2 mouse strains as both are commonly used to measure influenza disease. DBA/2 mice are notably susceptible to influenza virus infection, often displaying increased disease as compared to other mouse strains [17,30,31]. Intranasal infection with the GA/19 strain of swine influenza did not cause weight loss in either DBA/2 or BALB/c mice, although there were differences in weight between infected and naïve mice from each background, particularly among mice belonging to the DBA/2 group (Figure 3A). Despite the lack of weight loss, there was robust viral replication at two days post infection, with average lung titers from DBA/2 mice of 5.28 × 10^5^ pfu/mL and BALB/c mice at 2.64 × 10^4^ pfu/mL of lung homogenate. On day 4, titers had reduced significantly for DBA/2 mice to 8.32 × 10^4^ pfu/mL, while BALB/c mice virus titers at 2.74 × 10^4^ pfu/mL were not reduced (Figure 3B). At both timepoints, DBA/2 mice had significantly higher lung titers than BALB/c animals assayed at the same time. Pathological findings on day 5 showed mild pathology in lung tissues, regardless of genetic background. Interestingly, DBA/2 mice had a noted increased degree of necrotic bronchiolar epithelium and lymphocytic migration around vessels compared to BALB/c mice.

### 3.3. Viral Replication and Transmission in Swine

**Figure 4:**
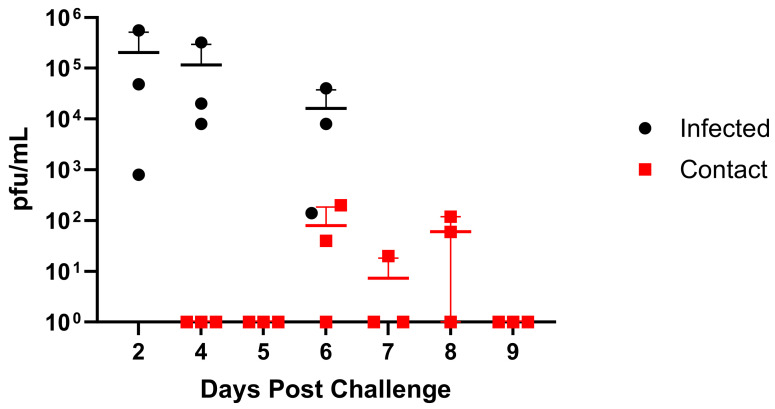
Replication and transmission of A/sw/GA/27480/19 (H1N2) in a swine model. Six-week-old pigs were inoculated intranasally with 2 × 10^6^ pfu of GA/19 in a 2 mL volume (n = 3). At 2, 4, and 6 dpi, BAL samples were collected (black data points). At 3 dpi, naïve contact animals (n = 3) were co-housed with infected animals, and nasal swabs were collected daily (red data points). Viral titers were determined by plaque assay. Error bars indicate mean ± SD.

The GA/19 virus was isolated from a Hampshire gilt, a 4 H show pig that developed a fever and died suddenly after travelling to an event [14,15], so we sought to assess GA/19 infection in healthy pigs. Clinical symptoms in infected pigs were mild. Viral titers in BAL fluid averaged 2.00 × 10^5^ pfu/mL on day 2 post-infection, decreasing slightly on day 4 post-infection to 1.56 × 10^5^ pfu/mL (Figure 4). The virus transmitted to two out of three contact pigs, as seen by shedding in nasal swabs (Figure 4). Three days after co-housing, nasal swabs from contact animals averaged 7.94 × 10^1^ pfu/mL and remained positive for viral shedding until day 6 post-contact. Nasal swab samples for infected and contact animals were also assayed for influenza virus load by qPCR, which showed that REU levels correspond to BAL titers among infected individuals (Figure S2). However, by this assay, all contact animals became positive by 3 days post-contact. All animals were positive for infection, as determined by serology as well. Despite rapid recovery, contact animals showed mild symptoms, limited to lethargy and elevated temperature.

### 3.4. Viral Replication and Transmission in Ferrets

**Figure 5:**
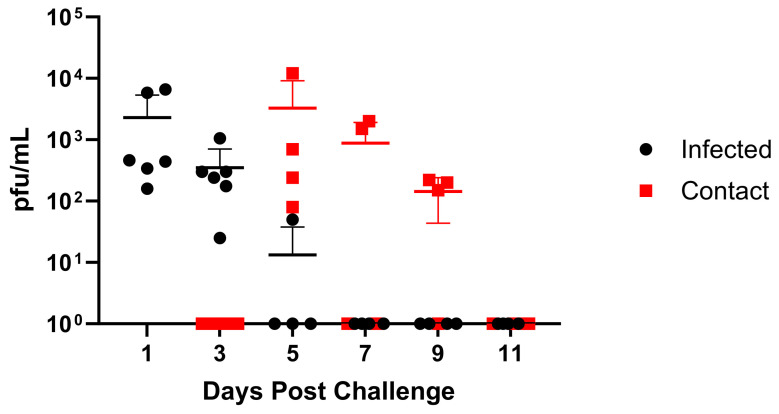
Replication and transmission of A/sw/GA/27480/19 (H1N2) in ferrets. Twelve-week-old ferrets were inoculated intranasally with 1 × 10^6^ pfu of GA/19 in a 1 mL volume (n = 6). At 1, 3, 5, 7, 9, and 11 dpi, nasal wash samples were collected, and titers were evaluated by plaque assay (black data points). At 2 dpi, naïve contact animals (n = 4) were co-housed with infected animals (1:1), and nasal washes were taken and evaluated for virus titer by plaque assay (red data points). Error bars indicate mean ± SD.

As ferrets are considered the “gold standard” animal model for human influenza virus infection [32], we assessed infection and clinical disease in this model. Ferrets infected with GA/19 showed the greatest viral titers in nasal washes at 1 dpi, with most animals clearing the virus by day 5, except for one animal remaining positive at this timepoint (Figure 5). Infection resulted in weight loss peaking at 3 dpi; however, this was mild, with average weights above baseline by 9 dpi (Figure S3). Contact ferrets introduced at 2 dpi were all positive for viral shedding by 5 dpi (day 3 post-contact (dpc)). Contact animals displayed a pattern of viral shedding similar to infected animals (Figure 5), with all animals negative in nasal washes by 11 dpi (8 dpc). Weight loss within the contact group was also mild but remained depressed through 11 dpi.

### 3.5. Viral Replication in In Vitro Substrates

**Figure 6:**
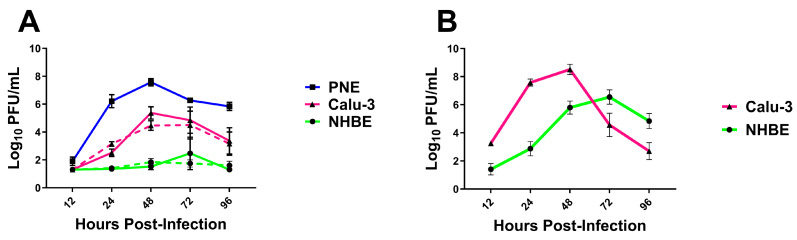
Replication of H1 influenza viruses in vitro. Primary porcine nasal epithelial (PNE), primary human bronchial epithelial (NHBE), and Calu-3 cells (all at ALI) were infected apically with either GA/19 (**A**) or A/CA/07/09 (**B**) at an MOI of 0.01. At 12, 24, 48, 72, and 96 h post-infection, the apical surface of cultures was washed, the fluid collected, and then titered for virus by plaque assay. Dashed lines denote duplicate experiments. Error bars indicate mean ± SD.

To further investigate the capability of GA/19 to replicate in human respiratory tissues, we cultured Calu-3 cells, normal human bronchial epithelial (NHBE) cells, and as a control, porcine nasal epithelial (PNE) cells, at an air–liquid interface (ALI). Upon differentiation and culture at ALI, all three cell substrates produced mucus and reflected the airway surface. The airway cell substrates were infected on the apical surface with GA/19 (MOI of 0.01), and the apical surface was sampled at 12 h intervals and then every 24 h to determine virus replication and differing levels of permissiveness to infection. Infection of PNE cells [25] resulted in the highest titers of GA/19, rapidly increasing to 1.62 × 10^6^ pfu/mL at 24 h post-inoculation and then peaking at 3.98 × 10^7^ pfu/mL by 48 h post-inoculation (Figure 6A). Calu-3 cells, derived from a human lung adenocarcinoma, are frequently used as a model of susceptibility to viral infection [33,34]. Our infections with the Calu-3 substrate with GA/19 showed rapid viral replication, reaching peak titers of 2.34 × 10^5^ pfu/mL by 48 h post-inoculation (Figure 6A). Surprisingly, infection of NHBE cells with the swine isolate showed contrasting results to the other cell substrates. Viral titer remained depressed compared to Calu-3 and PNE substrates at equivalent timepoints, never exceeding 1 × 10^3^ pfu/mL. Timing of peak viral titer was also retarded, being seen at 72 h post-inoculation as opposed to the observed 48 h for PNE and Calu-3 cells (Figure 6A). GA/19 virus replication kinetics were confirmed in Calu-3 and NHBE cells of a different donor background, with a duplicate experiment that demonstrated near identical virus titers over the 96 h assay (Figure 6A, dashed lines). While there was little to no virus replication, GA/19 was able to infect, as demonstrated by confocal imaging of virus NP antigen staining of NHBE cells at 96 h post-inoculation (Figure S4).

To confirm the permissiveness of the human cell substrates to a human influenza virus, we ran a complementary infection experiment with the 2009 pandemic virus, A/CA/07/09 (CA/09; MOI of 0.01). Calu-3 cells infected with CA/09 showed a pattern of replication kinetics markedly similar to that seen with GA/19, peaking at 48 h post-infection at 3.24 × 10^8^ pfu/mL (Figure 6B). NHBE cells infected with CA/09 also displayed a similar kinetics pattern to infections with GA/19, peaking at 72 h, but resulted in much greater titers at 24, 48, 72, and 96 h post-inoculation and a peak virus titer of 3.47 × 10^6^ pfu/mL by 72 h post-infection (Figure 6B), compared to the minimal levels seen with the swine-origin virus.

## 4. Discussion

Our studies show a notable discrepancy in outcomes of infection within the spectrum of models used to assess the pandemic risk of zoonotic influenza viruses. Utilizing specific elements within each of the three main criteria of the IRAT tool, we assessed the potential of the A/swine/Georgia/A19-27480/2019 (H1N2; GA/19) virus to impact human health. To investigate viral characteristics, we performed a phylogenetic analysis of sequences of both the HA and NA genes. This analysis demonstrated a close relationship of the GA/19 virus to contemporary swine H1-δ2 viruses within North America, as well as a surprisingly close relationship to human variant influenza isolates. Our studies further examined host interactions with the virus, utilizing several animal models to assess pathogenicity as well as transmission potential.

Every animal model of infection used showed unequivocal replication of the GA/19 virus, as well as transmission potential in both swine and ferret hosts. These results were corroborated by infection experiments in human and swine-derived airway epithelial cell culture models. The virus displayed similar growth kinetics in human Calu-3 and porcine nasal epithelial cells, peaking in both systems by 72 h post-infection, albeit with differing peak titers. Contradictory to these findings were the results of replication kinetics experiments in NHBE cells, a widely used model to ascertain the permissivity of human cells to viral infection [35]. Within this latter culture model, infection with the GA/19 virus resulted in little to no viral replication. Despite the lack of replication, confocal images confirmed viral invasion into the cell substrate by 96 h post-infection, suggesting that NHBEs were susceptible to infection, but the virus failed to replicate. Infection of the same NHBE cells with human-origin A/CA/07/2009 (pdmH1N1) confirmed them as permissive to IAV infection. Contrary to initial infections in NHBE cells, a repeat experiment using an alternate donor with varying demographics showed a modest capacity of the GA/19 virus for replication in human primary respiratory epithelial cells (Figure S5). The inter-donor variability of NHBE cells is a recognized problem in the context of influenza infection; however, the cause of this is likely multifactorial. Recent studies have demonstrated differential kinetics and amplitude of interferon gene expression subsequent to infection of NHBEs with human-origin H1N1 and H3N2 viruses. However, proteomic studies indicate that the problem may be even more nuanced. Here, the expression of proteins critical to the control of influenza virus replication, namely IFIT1, IFIT2, IFITM1, IFITM2, IFITM3, and MX, could have expression levels that vary as much as 10-fold between donors [36].

Infection of two strains of mice demonstrated ready replication of the GA/19 virus. Notably, not all influenza viruses readily infect mice without adaptation, and others will infect but replicate only to low titers and not elicit clinical disease [37]. We assessed GA/19 for infection and disease in two established models, the BALB/c and DBA/2, resistant and susceptible mouse strains, respectively. We observed robust replication but no clinical signs of disease or weight loss. However, we did note histopathological changes from infection and greater histopathology in the GA/19-infected DBA/2 mice. These results are consistent with previous studies demonstrating the enhanced susceptibility of DBA/2 mice to influenza infection [17,30,31].

Of the animal models used in our studies, ferrets, in particular, are considered the gold standard for influenza A virus infection, virulence, and transmissibility in humans, and are thus used in assays to determine the risks posed by avian, porcine, and emerging influenza A viruses [32,38,39,40]. The cellular ligand used by the influenza virus hemagglutinin to bind to and enter human cells, an α2,6-linked sialic acid, shows similar distributions in ferret and human respiratory tissues [38,41]. Additionally, influenza infection in ferrets has a clinical disease phenotype very similar to what is seen in humans, including weight loss, sneezing, and lethargy [40]. Our infection studies in the ferret model provided clear evidence of replication and transmission, a strong indication that the isolate would pose a threat to a human host.

Two different human-origin cell culture systems were used in our in vitro infections. Calu-3 cells are a continuous human epithelial lung cell line derived from a pulmonary adenocarcinoma and have been characterized extensively. They have been determined to display a sialic acid receptor profile that allows for infection with human influenza virus isolates [33,34]. Normal human bronchial epithelial cells are primary cells isolated from tracheobronchial tissue sections and have been used extensively in influenza research [35,41]. When cultured at an air–liquid interface, these cells display a mixed morphology, including ciliated and mucus-secreting goblet cells. Importantly, these cells share the same α2,6 sialic acid receptor profile as Calu-3 cells, a critical component in mammalian influenza virus entry to the host cell. Despite these similarities, our infection studies showed strikingly different results between the two substrates. This hints at a subtler set of factors distinguishing the two cell substrates, creating a more permissive environment in Calu-3 cells for replication of a swine-origin influenza virus. While both models serve as useful tools in studying influenza infection in human respiratory tissues, this disparity must be taken into consideration for future risk assessments of emerging swine influenza isolates.

There were several limitations present in our study. First, in our ferret infection studies, transmission was determined by placing naïve animals in direct contact with infected animals. More sophisticated housing systems have been created that would allow for the determination of potential virus transmission by airborne transmission in addition to contact transmission. Also, NHBE susceptibility to infection varied, likely based upon donor variability. The extent of this variability is not known and would require extensive screening to estimate the consistency of these substrates. Assessment of more contemporary human influenza A viruses in the in vitro infection models could strengthen confidence in the different cell culture models. Finally, our in vitro infections used primary human cells isolated from a single area of the respiratory tract. While the influenza virus is normally capable of infecting cells throughout the respiratory tract, the process normally begins in the nasal cavity and upper trachea. Including these tissues in future studies would provide greater insight into tissue-specific host restriction of swine influenza viruses.

The GA/19 virus was isolated from a 6-month-old show pig that developed a high fever and died suddenly after recently travelling to an event [14,15]. However, in our infection study, with high-health status 6-week-old pigs, we observed only mild clinical signs, despite robust replication in the lower and upper respiratory tract and efficient transmission to naïve contacts. Others have similarly observed mild clinical signs with other swine influenza viruses, following experimental infection [31,42,43]. While phylogenies among the gene segments reveal a genetically drifted variant from otherwise unremarkable swine influenza isolates, they do not explain the virulence of the initial case presentation from which the isolate was obtained. Histopathological findings from the dead gilt found influenza A antigen in the lung and lymphoid tissue, but bacterial infection (Streptococcus suis) was also detected in the lung. However, other viral infections (e.g., porcine circovirus, porcine reproductive, and respiratory syndrome virus) were excluded as predisposing factors [14]. Nonetheless, influenza A virus infection was determined to be the primary diagnosis, and the GA/19 isolate was highly related to other H1N2 virus infections across the Midwest, as well as two human H1N2v infections.

The detection of an influenza virus in a show pig with recent travel history is unsurprising. Surveillance of county and state fairs, as well as swine “jackpot shows”, suggests that more than one-third of county fairs and three-quarters of jackpot shows have influenza-infected pigs. Further, in one study, more than one-third of influenza-positive state fairs had >75% pigs test positive for influenza A virus [44]. Multiple studies have shown widespread influenza A virus infection in swine at fairs and shows, as well as dynamic evolution, transportation, and transmission of swine influenza viruses in North America [44,45,46,47,48,49]. These circulating swine influenza A viruses have resulted in significant numbers of human spillover infections [47,50,51,52]. As such, the relatedness of GA/19 to swine variant viruses isolated from humans [28,29] is similarly unsurprising and further supports surveillance and risk assessment activities.

In summary, we have confirmed the ability of GA/19 to replicate and transmit in multiple models of human influenza virus infection. Among these, our infection studies in mice and ferrets agree with the literature in their utility as models of infection with swine influenza A viruses. We have also characterized the extent to which the isolate replicates in swine, its native host species. Importantly, our experiments have highlighted considerations that must be taken into account when assessing the pandemic risk of influenza strains in vitro, as commonly used models of human respiratory infections possess widely differing abilities to host zoonotic influenza viruses.

## Data Availability

All sequences are publicly available in GenBank. The virus isolate sequenced for this study is listed under NCBI bioproject PRJNA600894, accession numbers PP554726, PP554727, PP554728, PP554729, PP554730, PP554731, PP554732, and PP558395.

## Supplementary Materials

The following supporting information can be downloaded at: https://www.mdpi.com/article/10.3390/v16040548/s1, Figure S1: Maximum likelihood phylogeny for swine isolates collected between 2014 and 2019; Figure S2: Nasal shedding of A/sw/GA/27480/19 (H1N2) in swine, as determined by qPCR; Figure S3: Weight loss in ferrets post-challenge with A/sw/GA/27480/19 (H1N2); Figure S4: Confocal image of NHBE cells infected with GA/19 at 96 h post-infection; Figure S5: Replication kinetics of GA/19 compared to CA/09 in NHBE cells from an alternate donor.
