# Supplementary material for: Pandemic Risk Assessment for Swine Influenza A Virus in Comparative In Vitro and In Vivo Models
Source: Viruses. 2024 Mar 31;16(4):548. doi: 10.3390/v16040548 (PMC11053818; doi:10.3390/v16040548)
Supplement: Supplementary file 1 [file viruses-16-00548-s001.zip › viruses-2919230-supplementary.pdf]

Figure S1

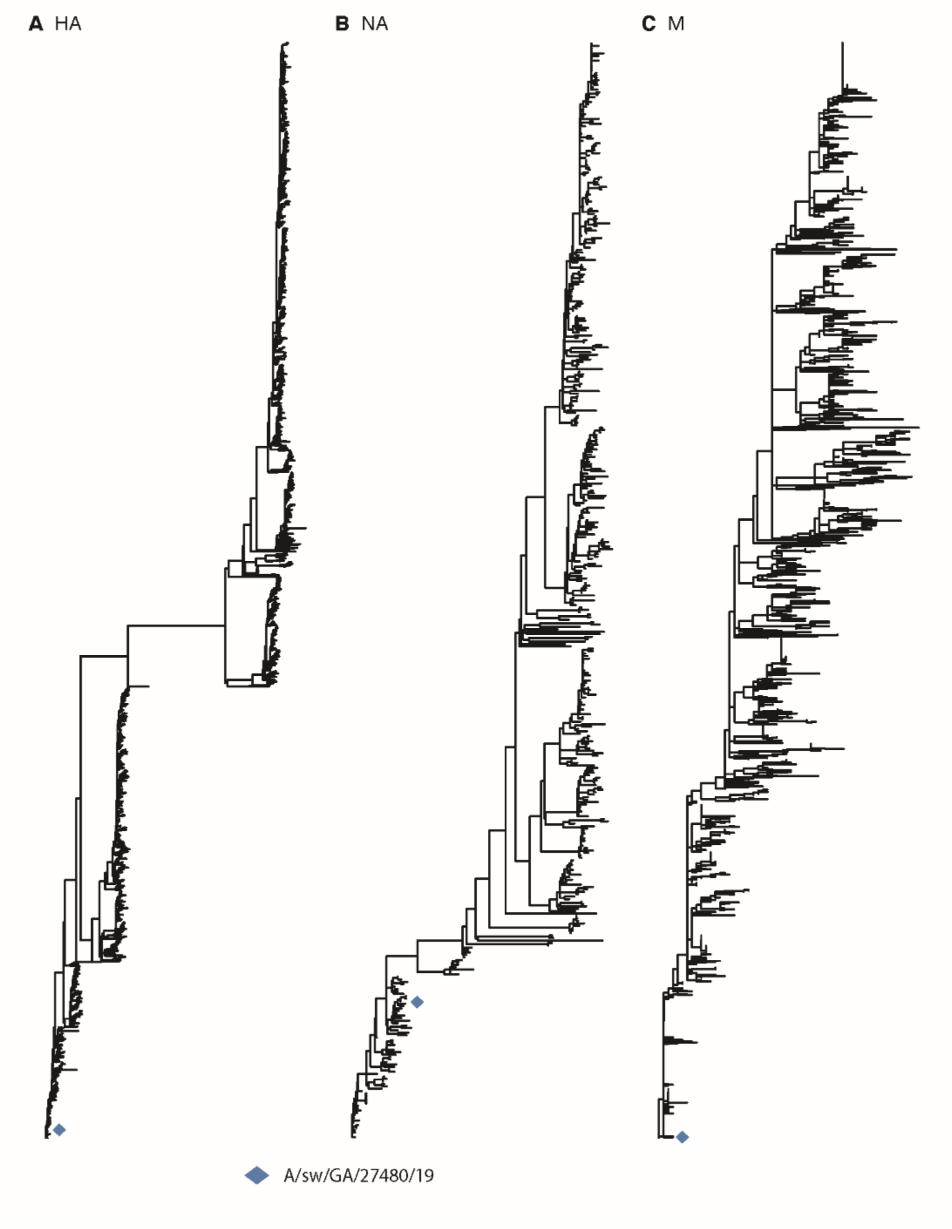

Figure S1. Maximum likelihood phylogeny for swine isolates collected between 2014 and 2019. Phylogenetic reconstructions for the hemagglutinin (A), neuraminidase (B) and matrix (C) genes within the context of U.S. swine influenza A isolates representing the 5 year period prior to isolation of the GA/19 virus.

Figure S2

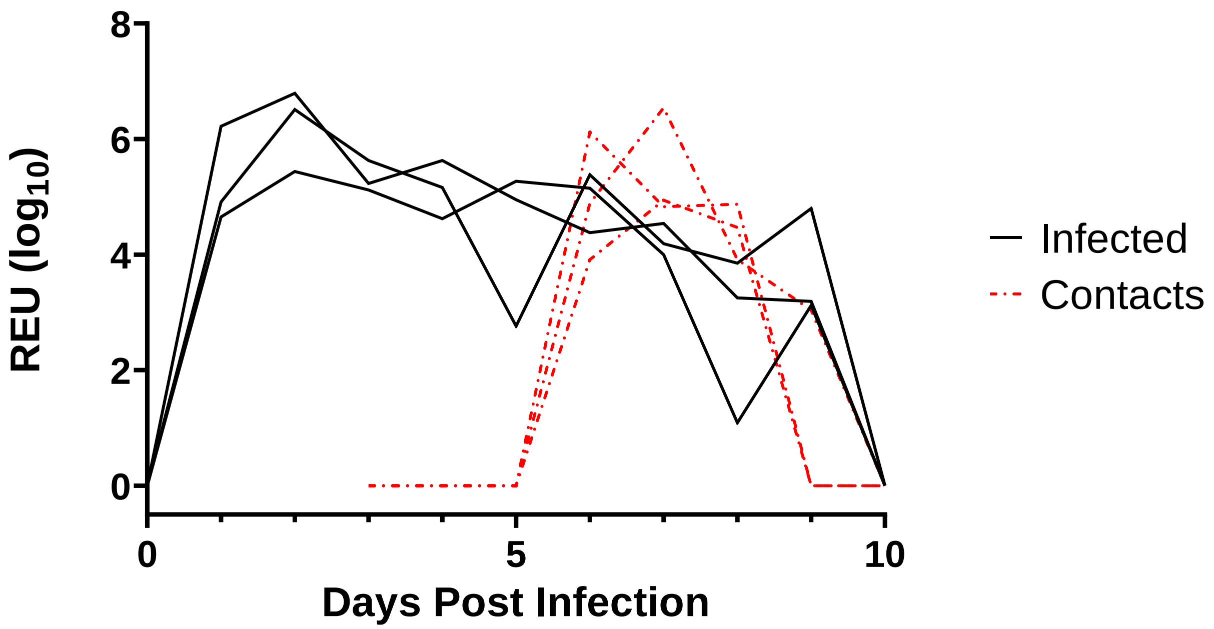

Figure S2. Nasal shedding of A/sw/GA/27480/19 (H1N2) in swine as determined by qPCR. Infected animals showed peak viral loads by 2 dpi, remaining positive until day 9. All three contact animals became positive by 3 dpi, two remaining positive until day 8 and one until day 9.

Figure S3

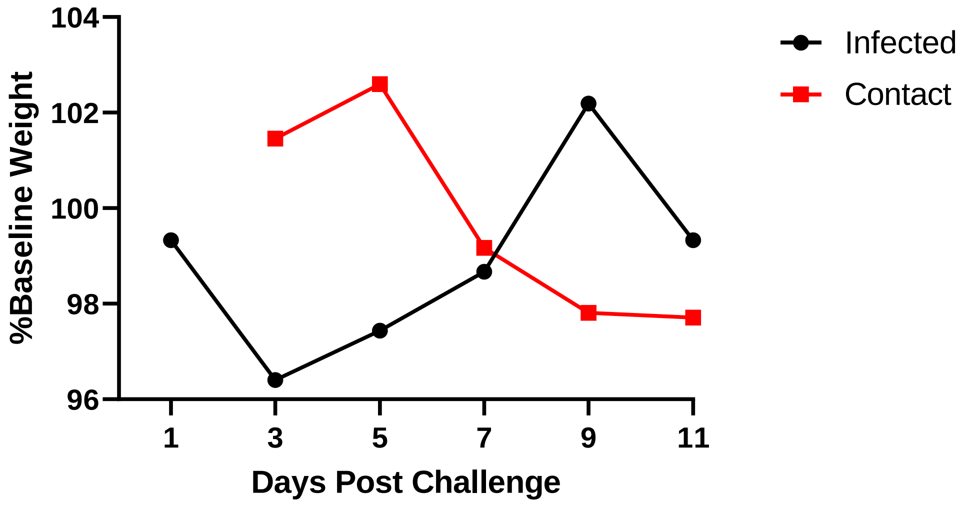

Fig**ure S3. Weight loss in ferrets post-challenge with A/sw/GA/27480/19 (H1N2).** Weight measurements were taken until 11 dpi. Infected animals experienced an initial decrease in weight before returning to baseline levels. Contact animals experienced a comparable decrease in weight corresponding to an onset of viral shedding.

Figure S4

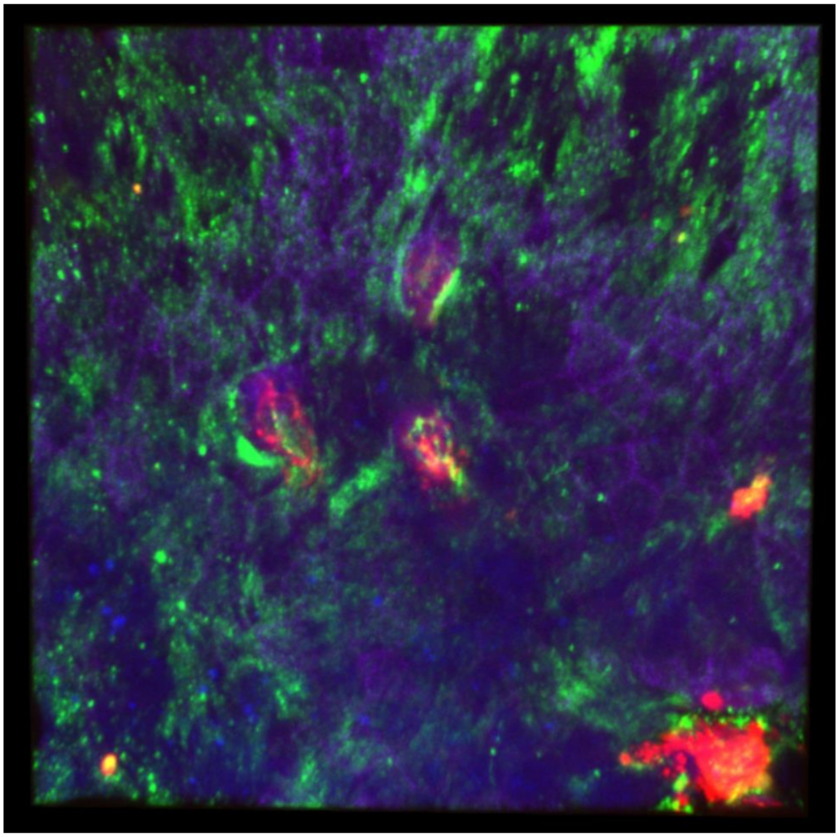

Figure S4. Confocal image of NHBE cells infected with GA/19 at 96 hours post-infection. Despite showing minimal viral replication, NHBE cultures infected with GA/19 showed clear evidence of viral invasion. Red foci indicate cells infected with the swine influenza isolate. Red: viral nucleoprotein, blue: nuclear stain, purple: F-actin, green: beta tubulin.

Figure S5

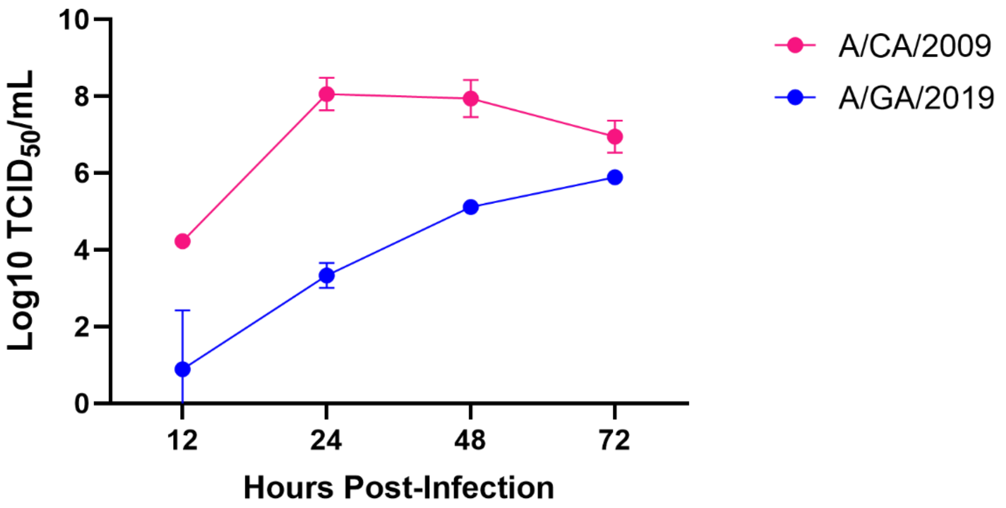

Experiment	Donor Sex	Donor Age	Donor Race	Doubling Time (hours)
1	M	56	C	27
2	F	66	B & H	31

Figure S5. Replication kinetics of GA/19 compared to CA/09 in an NHBE cells from an alternate donor. Cultures were infected apically with either GA/19 or CA/09 at an MOI of 0.001. At 12, 24, 48, and 72 hours post-infection the apical surface of cultures were washed and the fluid titered for virus by TCID50. Error bars indicate mean ± SD.
